# Discordant muscle-adipose phenotypes and insulin resistance in prediabetes: sex-specific associations and predictive utility of TyG-WHtR combined with HDL-C

**DOI:** 10.3389/fendo.2026.1937778

**Published:** 2026-10-15

**Authors:** Zhishan Wang, Chen Lin, Yuanya Xie, Liangliang Huang, Shaojian Xiao, Huiyun Chen, Hongjie Ge, Yuxin Zhou, Jiaying Chen, Wensi Wu, Xiaoling Wang, Shujiao Chen

**Affiliations:** 1 Department of Preventive Treatment of Disease, The Third Affiliated People’s Hospital of Fujian University of Traditional Chinese Medicine, Fuzhou, China; 2 Emergency Department, The Third Affiliated People’s Hospital of Fujian University of Traditional Chinese Medicine, Fuzhou, China; 3 College of Acupuncture and Tuina, Fujian University of Traditional Chinese Medicine, Fuzhou, China; 4 College of Traditional Chinese Medicine, Fujian University of Traditional Chinese Medicine, Fuzhou, China; 5 College of Rehabilitation Medicine, Fujian University of Traditional Chinese Medicine, Fuzhou, China

**Keywords:** prediabetes, insulin resistance, body composition, muscle-adipose phenotype, TyG-WHtR, HDL-C, nomogram

## Abstract

**Objective:**

We aimed to explore the interplay between muscle-adipose composite phenotypes and insulin resistance (IR) among individuals with prediabetes(Pre-DM), and to evaluate the discriminative capacity and sex-specific variations of the triglyceride-glucose-waist-to-height ratio (TyG-WHtR) when combined with high-density lipoprotein cholesterol (HDL-C).

**Methods:**

A total of 167 subjects with Pre-DM were enrolled and categorized into four phenotypes—high-muscle/low-fat, high-muscle/high-fat, low-muscle/high-fat, and low-muscle/low-fat—based on their appendicular skeletal muscle index and visceral fat level. Fasting blood glucose, lipid profiles, and body composition parameters were measured, and the TyG index and its derived indices were calculated. Hierarchical linear regression and logistic regression were used to evaluate the independent and interactive contributions of these phenotypes, lipid profiles, and TyG-derived indices to HOMA-IR. Subsequently, a nomogram was constructed using binary logistic regression, and its performance was evaluated via the area under the receiver operating characteristic curve (AUC), calibration curve, decision curve analysis (DCA), and clinical impact curve (CIC).

**Results:**

Significant intergroup differences emerged for body mass index, waist circumference, fasting insulin, HOMA-IR, HDL-C, and TyG-derived indices (all *P* < 0.05). Spearman’s correlation analysis revealed that fasting insulin and HOMA-IR were positively correlated with WHtR, waist circumference, BMI, body fat mass, visceral fat level, body fat percentage, and ASMI (all *P* < 0.01). Triglycerides showed a positive correlation with these metrics, whereas HDL-C exhibited a negative correlation (all *P* < 0.05). Notably, TyG-WHtR displayed the strongest correlation with HOMA-IR (*P* < 0.001). Hierarchical regression identified HM/HF as the strongest independent predictor of HOMA-IR, remaining significant after full adjustment (B = 3.264,*P* < 0.001). The protective effect of LM/LF was attenuated to marginal significance after adjusting for blood lipids (B=−0.705, *P* = 0.082), suggesting that this association largely reflects low visceral fat rather than muscle mass per se. The final model accounted for 36.0% of the variance in HOMA-IR. We did not detect a statistically significant multiplicative interaction (*P*=0.089); however, the study may be underpowered, and additive interaction was not formally assessed. In exploratory stratified analyses, the HM/HF effect was more pronounced in men (B = 5.662) and in adults aged <60 years (B = 4.425), whereas the protective LM/LF effect was restricted to women (B=−0.914). In the ≥60-year group, the sex coefficient changed from negative to positive without reaching significance, suggesting that age may modify the sex effect; formal interaction testing is needed to confirm this possibility. Logistic regression identified TyG-WHtR (OR=8.231) and HDL-C (OR=0.183) as independent determinants of IR; the corresponding Cohen’s d values exceeded the conventional large-effect threshold of 0.8. The combined nomogram achieved an AUC of 0.815 (95% CI 0.748–0.881). The Hosmer-Lemeshow test showed no evidence of departure from perfect calibration, and the calibration curve derived from 1000 bootstrap resamples closely followed the ideal diagonal line. Decision curve analysis indicated that, across threshold probabilities of 0.2–0.8, the net benefit remained consistently greater than that of both the “intervene-all” and “intervene-none” strategies.

**Conclusion:**

In Pre-DM, insulin resistance appears to be driven primarily by visceral adiposity rather than overall obesity. The metabolic impact of skeletal muscle is highly dependent on the surrounding lipid milieu. The interaction term between low muscle mass and high visceral fat was non-significant, with no multiplicative interaction detected. Consequently, it remained inconclusive whether their effects on IR followed an additive pattern. Sex and age may modify the association between body composition and IR, although formal interaction testing is needed to confirm this. The nomogram integrating TyG-WHtR and HDL-C demonstrated good calibration and may serve as a practical screening tool in primary care settings. Maintaining moderate muscle mass in the context of low visceral adiposity is likely to confer greater metabolic benefit.

## 1 Introduction

Insulin resistance (IR) constitutes the core pathophysiological basis of type 2 diabetes mellitus (T2DM) and is already pervasive during the prediabetic stage. Obesity, particularly visceral fat accumulation, is a well-established driver of IR. A dose-response meta-analysis encompassing 28 cohort studies demonstrated that each 1-unit increment in body mass index (BMI) was associated with a 4.6% increase in the risk of prediabetes (Pre-DM) (1). However, defining obesity solely by BMI overlooks the heterogeneity of body composition. Among overweight or obese middle-aged and older adults, Pre-DM is linked to diminished muscle endurance, lower appendicular muscle mass, and higher intramuscular lipid content, independent of total fat mass (2). Intramuscular fat deposition can impair insulin signaling pathways through inflammation and oxidative stress and further promote muscle atrophy; in turn, the reduction in muscle mass shrinks the glucose-disposal tissue pool, exacerbating IR and perpetuating a vicious cycle (3, 4). Recent evidence further indicates a higher prevalence of sarcopenia in the prediabetic population. For example, data from the U.S. National Health and Nutrition Examination Survey (NHANES) (5) show a sarcopenia prevalence of 11.8% in this population, compared with 5.4% in normoglycemic individuals.

Sarcopenia and obesity are often mutually reinforcing (6). Sarcopenic obesity, defined by the coexistence of low muscle mass and increased adiposity, could theoretically produce synergistic metabolic impairment, but the available evidence is inconsistent: some studies report the highest metabolic risk for the low-muscle/high-fat phenotype (7, 8), whereas others suggest that, in the absence of increased adiposity, low muscle mass may be accompanied by a lower risk of IR (9, 10). These inconsistencies likely arise from different diagnostic cut-points for muscle mass and adiposity, heterogeneity in study populations, and insufficient attention to interaction effects. Sex and age may further modify associations between muscle–fat phenotypes and IR, yet stratified data remain sparse. To address these gaps, among individuals with Pre-DM, we assessed body composition by bioelectrical impedance analysis, constructed a 2 × 2 muscle–fat classification based on muscle mass and visceral fat, and evaluated its association with IR using multiplicative interaction tests and sex- and age-stratified analyses. We then used multivariable logistic regression to identify independent predictors, aiming to inform individualized IR risk management.

## 2 Material and methods

### 2.1 Study design and patient selection

A total of 167 prediabetic adults, diagnosed in strict accordance with the criteria outlined in the Chinese Guidelines for the Prevention and Treatment of Type 2 Diabetes (2024 edition) (11), were enrolled. Inclusion criteria were as follows: (i) adult patients aged ≥18 years; (ii) fulfillment of the diagnostic thresholds for impaired fasting glucose (IFG) and/or impaired glucose tolerance (IGT) as stipulated in the same guideline (11); and (iii) voluntary participation with signed informed consent.

Exclusion criteria were as follows: (i) missing clinical data, inability to adhere to study procedures, or voluntary withdrawal; (ii) presence of diabetes mellitus or other endocrine disorders affecting glucose metabolism; (iii) severe psychiatric conditions (e.g., major depressive disorder, mania) or severe cognitive impairment rendering the individual unable to complete study assessments; (iv) severe cardiac, hepatic, or renal complications, other severe primary diseases, or mental or legal incapacity; and (v) lactating women, pregnant women, or women planning pregnancy.

Sample Size Estimation: We performed sample size estimation using G*Power (version 3.1.9.7) based on the R² increase method within the fixed model of multiple linear regression. The primary objective was to examine the association between four muscle-adipose phenotypes (using the high-muscle/low-fat phenotype as the reference) and the homeostasis model assessment of insulin resistance (HOMA-IR). Accordingly, we specified three primary predictors—the three dummy variables representing the four phenotypes. The model also adjusted for 12 covariates: age, sex, fasting blood glucose (FBG), 2-hour postprandial glucose (2hPG), glycated hemoglobin (HbA1c), triglycerides (TG), total cholesterol (TC), high-density lipoprotein cholesterol (HDL-C), low-density lipoprotein cholesterol (LDL-C), waist-to-height ratio (WHtR), visceral fat level (VFL), and appendicular skeletal muscle index (ASMI). This yielded a total of 15 predictors in the model (12 covariates plus 3 phenotype dummy variables). A priori sample size estimation was performed using G*Power 3.1.9.7 (F tests, linear multiple regression: fixed model, R² increase) with effect size f²=0.15, α=0.05, power=0.95, number of tested predictors=3, and total number of predictors=15. The minimum required sample size was 119. Our study ultimately enrolled 167 participants with Pre-DM, exceeding this minimum requirement and indicating that the overall analysis had sufficient statistical power to detect significant associations. We acknowledge that this sample size estimation pertains solely to the overall analysis; sex-stratified or subgroup analyses may have limited statistical power due to smaller sample sizes.

The study protocol was approved by the Institutional Review Board of the Third People’s Hospital of Fujian University of Traditional Chinese Medicine (Approval No. 2025KS-58-1) and adhered to the tenets of the Declaration of Helsinki. All participants provided written informed consent before inclusion in the study.

Body composition was assessed using an Inbody 570 bioelectrical impedance analyzer. VFL ≥10 was defined as high visceral adiposity, whereas a VFL of 1–9 was classified as normal visceral fat (12). Sex-specific cutoffs for the ASMI were derived from the median values within the study cohort. Low muscle mass was defined as ASMI <7.6 kg/m² in men and < 6.3kg/m² in women; normal/high muscle mass was defined as ASMI ≥7.6 kg/m² in men and ≥ 6.3kg/m² in women. On the basis of these criteria, participants were categorized into four groups, (**Table 1**).

**Table 1:** Classification of participants based on ASMI and VFL status.

Item	Low visceral fat (VFL < 10)	High visceral fat (VFL ≥ 10)
High ASMI (≥ median)	High Muscle/Low Fat(HM/LF)	High Muscle/High Fat(HM/HF)
Low ASMI (< median)	Low muscle-low fat(LM/LF)	Low muscle-high fat(LM/HF)

### 2.2 Data acquisition

Blood Biochemistry Analysis: Fasting blood samples were collected from all participants at the Department of Clinical Laboratory, The Third People’s Hospital of Fujian University of Traditional Chinese Medicine. Participants were required to fast for 8–10 hours overnight prior to venipuncture. Key metabolic parameters were measured, including FBG,2hPG, HbA1c, fasting insulin (FIns), TG, TC, HDL-C and LDL-C. Based on these measurements, several insulin resistance-related indices were subsequently calculated: the HOMA-IR, triglyceride-glucose product (TyG), TyG index combined with body mass index (TyG-BMI), TyG index combined with waist circumference (TyG-WC), and TyG index combined with waist-to-height ratio (TyG-WHtR).

Body Composition Measurement: Whole-body composition was assessed using an InBody 570 device (Biospace Co., Ltd., Seoul, Korea). Prior to the measurement, participants were instructed to fast, void their bladder and bowels, refrain from strenuous physical activity, and stand quietly for 5 minutes. They wore lightweight hospital gowns and removed all items from their pockets. After height measurement, participants removed their shoes and socks, and electrode sites were cleaned with conductive wet wipes. During the test, participants stood barefoot on the foot electrodes, aligning their feet with the electrode contours, while holding the hand electrodes with arms naturally hanging apart from the torso. The device measured various metrics, including body weight, BMI, skeletal muscle mass (SMM), VFL, body fat mass (BFM), percentage of body fat (PBF), basal metabolic rate (BMR), WHtR, and the ASMI.

### 2.3 Statistical analysis

The following statistical analyses were performed in accordance with the nature of the data. All analyses were conducted using SPSS 26.0, supplemented by R version 4.4.1 where indicated. Continuous variables are presented as mean ± standard deviation if normally distributed, or as median (first quartile [Q1], third quartile [Q3]) if non-normally distributed.

Continuous variables were compared among the four groups using the Kruskal-Wallis H test, and categorical variables were compared using the chi-square test or Fisher’s exact test, as appropriate. Spearman correlation analysis was used to examine bivariate associations of body composition parameters and metabolic indices with HOMA-IR, providing a basis for variable selection in subsequent multivariable modeling. Hierarchical linear regression models were constructed with continuous HOMA-IR as the dependent variable. Model 1 included body composition phenotypes (with the high-muscle/low-fat subtype as the reference). Model 2 was further adjusted for age and sex, and Model 3 additionally included TG, HDL-C, and LDL-C. The independent contribution of each block of variables to the variation in HOMA-IR was assessed using ΔR².To examine effect modification by sex and age, the hierarchical regression analyses were repeated separately in male and female subgroups and in subgroups aged <60 and ≥60 years. Clinical insulin resistance was defined as HOMA-IR ≥2.5. Candidate variables with *P* < 0.05 in univariate analyses were entered into multivariable logistic regression; after excluding variables with variance inflation factor (VIF) ≥5, forward stepwise selection based on the likelihood ratio was used to identify independent predictors, and odds ratios (ORs) with 95% confidence intervals (CIs) were calculated. Receiver operating characteristic (ROC) curve analysis was used to evaluate the predictive performance of TyG-WHtR, HDL-C, and their combined model, with discrimination quantified by the area under the curve (AUC). Calibration was assessed using the Hosmer-Lemeshow test, calibration curves, and Brier scores, with internal validation via 1,000 bootstrap resamples. Clinical utility and net benefit were evaluated using decision curve analysis (DCA) and clinical impact curves (CIC). We also compared associated factors between the IR and non-IR groups, calculated Cohen’s d effect sizes, and interpreted the strength of associations according to Cohen’s conventions (0.2, 0.5, and 0.8 representing small, medium, and large effects, respectively).All statistical analyses were performed using SPSS 26.0 and R 4.4.1, and a two-sided *P* value <0.05 was considered statistically significant.

## 3 Results

### 3.1 Demographics and disease characteristics

A total of 167 prediabetic participants were enrolled. In the HM/LF group, the mean age was 57.00(48.00,63.00) years and 28 men (39.44%) were included; the HM/HF group had a mean age of 56.00(41.00,68.00) years, with 8 men (34.78%); the LM/HF group had a mean age of 53.50(42.00,65.75) years and consisted entirely of 12 women (100.00%); and the LM/LF group had a mean age of 58.00(53.50,64.00) years, with 25 men (40.98%). No statistically significant differences in age or height were detected across the four groups (*P*>0.05). Normality testing revealed that the majority of continuous variables were non-normally distributed within each group; accordingly, the Kruskal–Wallis H test was uniformly applied for all four-group comparisons. Data are reported as mean ± SD or median (Q1, Q3), with the significance level set at α=0.05. See **Table 2**.

**Table 2:** Baseline characteristics of the study population by body composition phenotype (Mean ± SD/Median (Q1, Q3)).

Variable	HM/LF	HM/HF	LM/HF	LM/LF	H	*P*	Pairwise comparisons (Adjusted *P*value)
Sex (male/female)	28/43	8/15	0/12	25/36	–	–	–
Age (years)	57.00(48.00,63.00)	56.00(41.00,68.00)	53.50(42.00,65.75)	58.00(53.50,64.00)	3.428	0.330	–
Height (cm)	161.00(155.00,170.00)	160.00(155.00,170.00)	159.00(152.75,162.25)	160.00(155.00,166.50)	3.893	0.273	–
Weight (kg)	61.70(57.20,70.10)	75.20(64.00,91.30)	59.60(56.05,64.60)	55.00(50.40,63.55)	46.594	<0.001	G4<G3<G1<G2^1^

G1=HM/LF; G2=HM/HF; G3=LM/HF; G4=LM/LF. Data are median (Q1, Q3). Kruskal-Wallis H test with Dunn’s *post hoc* test (Bonferroni-adjusted): G4 vs G1/G2 (*P* < 0.001); G1 vs G2 (*P* = 0.008); G2 vs G3 (*P* = 0.022).

### 3.2 Correlations between body composition, metabolic parameters, and IR

To investigate the associations between body composition, metabolic parameters, and IR, we conducted Spearman correlation analysis. FIns and HOMA-IR were positively correlated with all obesity-related indices—including WHtR, WC, BMI, BFM, VFL, and PBF—as well as with ASMI (all *P* < 0.01). Within the lipid profile, TG showed positive correlations with these obesity and muscle measures, whereas HDL-C displayed negative correlations (all *P* < 0.05). Among the TyG index and its derived indices, TyG-WHtR exhibited the strongest correlation with HOMA-IR, followed by TyG-BMI and TyG-WC; all three correlations were significantly stronger than that of the TyG index alone (all *P* < 0.001). The complete correlation matrices are provided in **Supplementary Figures S1**-**S4**.

### 3.3 Metabolic stratification defined by muscle-adipose phenotypes

To dissect the heterogeneity of body composition more precisely, we classified participants into four phenotypes based on ASMI and VFL: HM/LF, HM/HF, LM/HF, and LM/LF. Intergroup comparisons revealed significant differences across the four groups in WC, BMI, FIns, HOMA-IR, HDL-C, and all TyG-derived indices (all *P* < 0.05). Regarding the metabolic health profile, the LM/LF group exhibited the most favorable status, with the lowest levels of HOMA-IR, FIns, and TyG-derived indices. Conversely, the HM/HF group carried the heaviest metabolic burden, displaying the highest levels of FBG, TyG, and TyG-BMI. Detailed pairwise comparison results and statistics are provided in **Supplementary Tables S1**-**S3**.

### 3.4 Stratified quantitative relationships between body composition, blood lipids, and insulin resistance

#### 3.4.1 Independent contributions and effect modification

To evaluate the independent contributions of muscle-adipose phenotypes and blood lipid indices to IR, we constructed a hierarchical linear regression model with continuous HOMA-IR as the dependent variable. Variables were entered sequentially based on *a priori* causal hierarchy: body composition phenotypes (reference: HM/LF),age and sex, TG, HDL-C, and LDL-C. In Model 1, body composition phenotypes alone accounted for 26.8% of the total variance in HOMA-IR (*P* < 0.001). The HM/HF phenotype showed a strong positive association with HOMA-IR (B = 3.409, 95% CI 2.290–4.529, *P* < 0.001), whereas the LM/LF phenotype was inversely associated (B=−0.984, 95% CI −1.799 to −0.169, *P* = 0.018). After adjustment for age and sex (Model 2), the direction of these associations remained unchanged: the HM/HF phenotype remained significantly associated with higher HOMA-IR (B = 3.492, *P* < 0.001), and the LM/LF phenotype with lower HOMA-IR (B=−0.857, *P* = 0.036), with the model explaining 32% of the variance (ΔR²=0.052, *P* = 0.003). Further inclusion of lipid parameters (Model 3) modestly attenuated the independent effect of the HM/HF phenotype, which remained highly significant (B = 3.264, 95% CI 2.172–4.356, *P* < 0.001). In contrast, the protective effect of the LM/LF phenotype weakened to a marginal level (B=−0.705, *P* = 0.082), suggesting that its metabolic benefit may be partly attributable to a more favorable lipid profile. Age and triglycerides were also retained in the final model: each additional year of age was associated with a 0.033 decrease in HOMA-IR (*P* = 0.032), and each 1-unit increase in triglycerides was associated with a 0.445 increase in HOMA-IR (*P* = 0.047). The final model explained 36.0% of the total variance in HOMA-IR (**Table 3A**).

**Table 3A:** Hierarchical linear regression: Body composition and lipids as predictors of IR.

Variable	Step 1B(95%CI)	SE	t	Step 2B(95%CI)	SE	t	Step 3B(95%CI)	SE	t
Constant	3.597 (3.044~ 4.151)	0.280	12.826***	6.134 (4.329~ 7.939)	0.914	6.713***	6.608 (3.753~9.464)	1.446	4.571***
Low muscle-low fat	−0.984(−1.799~ −0.169)	0.413	−2.385^*^	−0.857 (−1.656~ −0.057)	0.405	−2.116^*^	-0.705 (-1.499~0.089)	0.402	-1.753
High muscle-high fat​	3.409 (2.290~ 4.529)	0.567	6.013^***^	3.492 (2.405~ 4.578)	0.550	6.346^***^	3.264 (2.172~4.356)	0.553	5.904***
Low muscle-high fat	1.033 (−0.423~ 2.490)	0.738	1.401	1.415 (−0.027~ 2.858)	0.730	1.937	0.996 (-0.442~2.434)	0.728	1.368
Age	—	—	—	−0.036 (−0.066~ −0.005)	0.016	−2.285^*^	-0.033 (-0.064~-0.003)	0.015	-2.159*
Sex ​	—	—	—	−1.002 (−1.747~ −0.257)	0.377	−2.658^**^	-0.646 (-1.430~0.137)	0.397	-1.629
LDL-C	—	—	—	—	—	—	-0.077 (-0.609~0.455)	0.269	-0.285
HDL-C	—	—	—	—	—	—	-1.003 (-2.404~0.397)	0.709	-1.415
TG	—	—	—	—	—	—	0.445 (0.007~0.883)	0.222	2.005*

B represents unstandardized coefficients; SE denotes standard error; 95% CI indicates 95% confidence interval. The dependent variable was continuous homeostasis model assessment of insulin resistance (HOMA-IR). Low muscle–low fat, high muscle–high fat, and low muscle–high fat were binary dummy variables (1= yes, 0= no), with the high muscle–low fat group serving as the reference. Sex was coded as 1= male, 0= female. Step 1:R²=0.268;Step 2:ΔR²=0.052,*P* = 0.003;Step 3:ΔR²=0.040, *P* = 0.022; *P < 0.05,**P < 0.01, ***P < 0.001.

#### 3.4.2 Independent and additive effects

To further examine the potential multiplicative interaction between low muscle mass and high visceral fat on HOMA-IR, a product term was added to the main-effects model (**Table 3B**). The interaction term did not reach statistical significance (B=−1.563, 95% CI: −3.368 to 0.241, *P* = 0.089), and the model’s explanatory power increased by only 0.012 (ΔR²=0.012), providing no support for a multiplicative joint effect of these two factors on HOMA-IR. When interaction terms were included, the effect of high visceral fat was further amplified, rising from 2.724 in Model 1 to 3.264 (*P* < 0.001). By contrast, the main effect of low muscle mass became non-significant (B= −0.705,*P* = 0.082).Age and triglycerides remained significantly associated with HOMA-IR (age: B=−0.033, *P* = 0.032; TG: B = 0.445, *P* = 0.047). These findings suggest that the inverse association between low muscle mass and lower HOMA-IR may be driven primarily by confounding due to low visceral fat, rather than reflecting an independent protective effect of low muscle mass per se.

**Table 3B:** Multiple linear regression: Muscle and fat mass (with interaction) and lipids as predictors of IR.

Variable	Model 1B(95%CI)	SE	t	Model 2B(95%CI)	SE	t
Constant	6.538 (3.667~9.410)	1.454	4.497***	6.608(3.753~9.464)	1.446	4.571***
High fat mass	2.724 (1.822~3.626)	0.457	5.965***	3.264 (2.172~4.356)	0.553	5.904***
Low muscle mass	-1.013 (-1.727~-0.298)	0.362	-2.800**	-0.705 (-1.499~0.089)	0.402	-1.753
Age	-0.031(-0.062~0.000)	0.015	-1.994*	-0.033 (-0.064~-0.003)	0.015	-2.159*
Sex	-0.758(-1.536~0.019)	0.394	-1.927	-0.646 (-1.430~0.137)	0.397	-1.629
TG	0.406 (-0.032~0.845)	0.222	1.83	0.445 (0.007~0.883)	0.222	2.005*
HDL-C	-0.952(-2.360~0.455)	0.713	-1.336	-1.003 (-2.404~0.397)	0.709	-1.415
LDL-C	-0.032 (-0.565~0.500)	0.27	-0.12	-0.077 (-0.609~0.455)	0.269	-0.285
Low muscle mass×high visceral fat (interaction)	—	—	—	-1.563 (-3.368~0.241)	0.914	-1.711
R²	0.349			0.36		
ΔR²	—			0.012		
F change(*P*)	12.158(<0.001)			11.133(<0.001)		

The dependent variable was continuous HOMA-IR. High visceral fat was coded as 1 for VFL ≥ grade 10 and 0 for VFL < grade 10. Low muscle mass was coded as 1 when ASMI was below the sex-specific median and 0 otherwise. Sex was coded as 1=male and 0=female. Model 1 was the main effects model, and Model 2 incorporated the low muscle mass × high visceral fat interaction term into Model 1. TG: triglycerides; HDL-C: high-density lipoprotein cholesterol; LDL-C: low-density lipoprotein cholesterol.*P < 0.05,**P < 0.01, ***P < 0.001.

#### 3.4.3 Differential modifying effects of sex and age

Exploratory stratified analyses revealed differential influences of sex and age on the associations between body composition phenotypes and IR (**Tables 3C**, **3D**).

**Table 3C:** Multiple linear regression model of IR stratified by sex.

Variable	male(n=61)	SE	t	female(n=106)	SE	t
	B(95%CI)			B(95%CI)		
Constant	10.208 (4.348∼16.068)	2.923	3.492**	3.302(0.389∼6.214)	1.468	2.249*
Age	-0.063 (-0.127∼0.001)	0.032	-1.975	-0.007 (-0.038∼0.024)	0.015	-0.448
TG	0.236 (-0.497∼0.968)	0.365	0.645	0.517 (-0.041∼1.075)	0.281	1.840
HDL-C	-2.509 (-6.133∼1.115)	1.808	-1.388	-0.587 (-1.921∼0.747)	0.672	-0.873
LDL-C	-0.191 (-1.306∼0.924)	0.556	-0.344	0.212 (-0.303∼0.727)	0.260	0.816
High muscle-high fat	5.662(3.350∼7.975)	1.153	4.910***	1.701(0.656∼2.746)	0.527	3.230**
Low muscle-high fat​	—	—	—	0.710 (-0.422∼1.843)	0.571	1.244
Low muscle-low fat	-0.352 (-1.940∼1.235)	0.792	-0.445	-0.914* (-1.712∼-0.115)	0.402	-2.271
R²	0.471			0.350		
ΔR²	0.412			0.304		
F change(*P*)	8.014(<0.001)			7.54(<0.001)		

The dependent variable was continuous HOMA-IR. The reference group was the high muscle–low fat group. The low muscle–high fat phenotype was absent in men, and therefore this variable was not included in the male model. TG: triglycerides;HDL-C: high-density lipoprotein cholesterol; LDL-C: low-density lipoprotein cholesterol.*P < 0.05,**P < 0.01, ***P < 0.001.

**Table 3D:** Multiple linear regression model of IR stratified by age.

Variable	<60years (n=106)	SE	t	≥60years (n=61)	SE	t
	B(95%CI)			B(95%CI)		
Constant	5.598(2.306∼ 8.889)	1.659	3.375**	4.102 (1.441∼ 6.763)	1.327	3.092**
TG	0.187 (-0.396∼0.770)	0.294	0.637	0.545 (0.007∼1.083)	0.268	2.033*
HDL-C	-1.420 (-3.343∼0.504)	0.969	-1.465	-1.097 (-2.629∼0.435)	0.764	-1.436
LDL-C	0.085 (-0.695∼0.865)	0.393	0.215	-0.202 (-0.719∼0.315)	0.258	-0.785
High muscle-high fat	4.425 (2.912∼5.938)	0.763	5.803***	0.651 (-0.488∼1.789)	0.568	1.146
Low muscle-high fat	1.068 (-1.083∼3.218)	1.084	0.985	1.202 (-0.109∼2.513)	0.654	1.838
Low muscle-low fat	-0.926 (-2.035∼0.184)	0.559	-1.655	-0.299 (-1.153∼0.555)	0.426	-0.702
SEX(female=1)	-1.292 (-2.381∼-0.204)	0.549	-2.356*	0.676 (-0.156∼1.508)	0.415	1.631
R²	0.436			0.339		
ΔR²	0.396			0.252		
F change(*P*)	10.831(<0.001)			3.884(0.002)		

The dependent variable was continuous HOMA-IR. The high muscle–low fat group served as the reference. TG: triglycerides;HDL-C: high-density lipoprotein cholesterol; LDL-C: low-density lipoprotein cholesterol.*P < 0.05,**P < 0.01, ***P < 0.001.

Sex-stratified analyses revealed sexual dimorphism in IR determinants. The model explained a greater proportion of variance in men (ΔR²= 0.412,*P*<0.001) than in women (ΔR² =0.304, *P*<0.001). HM/HF exhibited a robust independent positive association in men (B=5.662, *P*<0.001), which was markedly attenuated in women (B=1.701, *P*=0.002), suggesting sex-specific metabolic consequences of this phenotype. Conversely,LM/LF conferred an independent protective effect exclusively in women (B=-0.914,*P*=0.025). 

Age-stratified analyses indicated age-dependent modifications in the relationships between body composition phenotypes and IR. Among participants younger than 60 years, HM/HF was strongly associated with increased IR (B = 4.425, *P* < 0.001), whereas female sex correlated with lower HOMA-IR (B=−1.292, *P* = 0.020). LM/LF exhibited a negative trend that did not reach statistical significance (*P* = 0.101), and conventional lipid indices showed no independent contribution. In individuals aged 60 years or older, the association with HM/HF was no longer significant. Instead, TG emerged as the only significant positive predictor (B = 0.545, *P* = 0.047), and the sex disparity vanished entirely (*P* = 0.109). Notably, the sex coefficient in the ≥60 age group reversed direction—shifting from negative to positive—although this trend did not achieve statistical significance.

### 3.5 Multivariable logistic regression analysis of insulin resistance

While hierarchical regression quantified the contributions of various factors to the continuous variation in HOMA-IR, logistic regression provides ORs—a more clinically interpretable effect metric. By identifying robust independent predictors through stepwise selection, this approach informs variable selection for the subsequent nomogram model. Building on the continuous analysis, we further dichotomized insulin resistance using a HOMA-IR cutoff of 2.5 to create a binary clinical outcome. Multivariable logistic regression was then performed to identify factors independently associated with this outcome. In this analysis, IR status (HOMA-IR ≥2.5 vs. <2.5) served as the dependent variable. Independent variables included those with *P* < 0.05 in the univariable logistic regression, along with clinical covariates such as sex and age (**Table 4A**). After collinearity diagnostics, the VIF for all retained variables were <5, well below the conventional threshold of 10, indicating no substantial multicollinearity.

**Table 4A:** Univariate logistic regression analysis of influencing factors for IR.

Variable	β	SE	Waldχ²	*P*	OR	95%CI
BMR	0.001	0.001	1.610	0.205	1.001	0.999 ~ 1.003
SMI	0.550	0.201	7.514	0.006	1.734	1.170 ~ 2.570
WHtR​	23.547	5.422	18.864	<0.001	1.684×10¹^0^	4.088×10^5^ ~ 6.936×10¹^4^
2hPG	0.055	0.056	0.964	0.326	1.056	0.947 ~ 1.179
HbA1c	0.290	0.202	2.073	0.150	1.337	0.900 ~ 1.985
TG	1.421	0.364	15.201	<0.001	4.140	2.027 ~ 8.455
TC	-0.023	0.181	0.016	0.898	0.977	0.686 ~ 1.392
HDL-C	-2.983	0.654	20.792	<0.001	0.051	0.014 ~ 0.183
LDL-C	0.445	0.246	3.285	0.070	1.561	0.964 ~ 2.527
BFM	0.181	0.047	14.773	<0.001	1.198	1.093 ~ 1.314
PBF	0.101	0.031	10.361	0.001	1.107	1.040 ~ 1.177
VFL​	0.254	0.076	11.078	0.001	1.289	1.110 ~ 1.496
SMM	0.059	0.035	2.762	0.097	1.061	0.989 ~ 1.137
Age​	-0.013	0.015	0.768	0.381	0.987	0.959 ~ 1.016
BMI	0.347	0.080	18.882	<0.001	1.414	1.210 ~ 1.654
Sex	-0.411	0.355	1.337	0.248	0.663	0.330 ~ 1.330
FBG	0.538	0.173	9.676	0.002	1.713	1.220 ~ 2.405
Fins	2.063	0.458	20.264	<0.001	7.867	3.205 ~ 19.312
TyG	1.954	0.401	23.715	<0.001	7.059	3.215 ~ 15.500
TyG-BMI	0.047	0.009	26.246	<0.001	1.048	1.029 ~ 1.067
TyG-WHtR	2.524	0.492	26.333	<0.001	12.476	4.758 ~ 32.711
TyG-WC	0.013	0.003	22.699	<0.001	1.013	1.008 ~ 1.019

We performed forward stepwise logistic regression (likelihood ratio method) to evaluate variables including SMI, TyG-WHtR, HDL-C, VFL, age, and sex. Variables with *P* < 0.05 were retained to construct the final multivariable logistic regression model (**Table 4B**). The multivariable logistic regression analysis revealed that TyG-WHtR was an independent risk factor for IR (OR = 8.231, 95% CI:2.998–22.602, *P* < 0.001). For each 1-unit increase in TyG-WHtR, the risk of IR increased by approximately 7.2-fold. HDL-C was a protective factor (OR = 0.183,95% CI:0.044–0.765,*P* = 0.020), with each 1-unit increase associated with an approximately 81.7% reduction in IR risk. The overall model was statistically significant (Omnibus χ²=49.291,*P* < 0.001). The Hosmer-Lemeshow test yielded *P* = 0.650, suggesting a good fit of the model. The model’s explanatory power was indicated by Cox & Snell R²=0.256 and Nagelkerke R²=0.358. The overall classification accuracy was 76.6% (**Table 4C**).

**Table 4B:** Multicollinearity diagnostics of the variables.

Variable	B	SE	β	*P*	VIF
Constant	–0.405	0.541	—	0.455	—
VFL	–0.033	0.018	–0.224	0.072	3.32
ASMI	–5.93×10^-5^	0.055	0	0.999	2.65
HDL-C	–0.326	0.13	–0.207	0.013	1.48
TyG-WHtR	0.394	0.102	0.539	<0.001	4.22
Age	–0.001	0.003	–0.013	0.853	1.04
Sex	–0.005	0.103	–0.006	0.959	2.45

**Table 4C:** Multivariable logistic regression analysis of influencing factors for IR.

Item	β	SE	Wald	*P*	OR	95%CI
TyG-WHtR	2.108	0.515	16.729	<0.001	8.231	2.998~22.602
HDL-C	–1.699	0.730	5.417	0.020	0.183	0.044~0.765
Constant	–6.301	2.648	5.664	0.017	0.002	—

### 3.6 Development and accuracy assessment of the nomogram prediction model

#### 3.6.1 Construction of the nomogram

Independent predictors of IR risk in Pre-DM were identified via multivariable logistic regression and integrated into a nomogram developed with the rms package in R. To facilitate clinical application, raw regression coefficients were rescaled to a 0–100 point scoring system. Specifically, TyG-WHtR—the predictor with the widest effect range—was assigned the maximum score of 100 points across its value range, while HDL-C scores were allocated proportionally based on its regression coefficient relative to TyG-WHtR. The total score axis (0–120 points) maintains a strict linear relationship with the linear predictor of the original regression equation (adjusted for the intercept) and maps linearly to the IR risk probability axis (0.1 to 0.95) at the bottom of the nomogram.

Because HDL-C serves as a protective factor against IR (negative regression coefficient, B=-1.699), reverse coding was applied to preserve the consistent logic that higher scores correspond to higher IR risk. Consequently, the HDL-C axis on the nomogram is displayed in descending order (2.2 to 0.6 mmol/L). In contrast, TyG-WHtR, a positive predictor, exhibits increasing scores with increasing values. The units and effect directions of all axes in the nomogram align strictly with the final regression equation (**Figure 1**).

**Figure 1:**
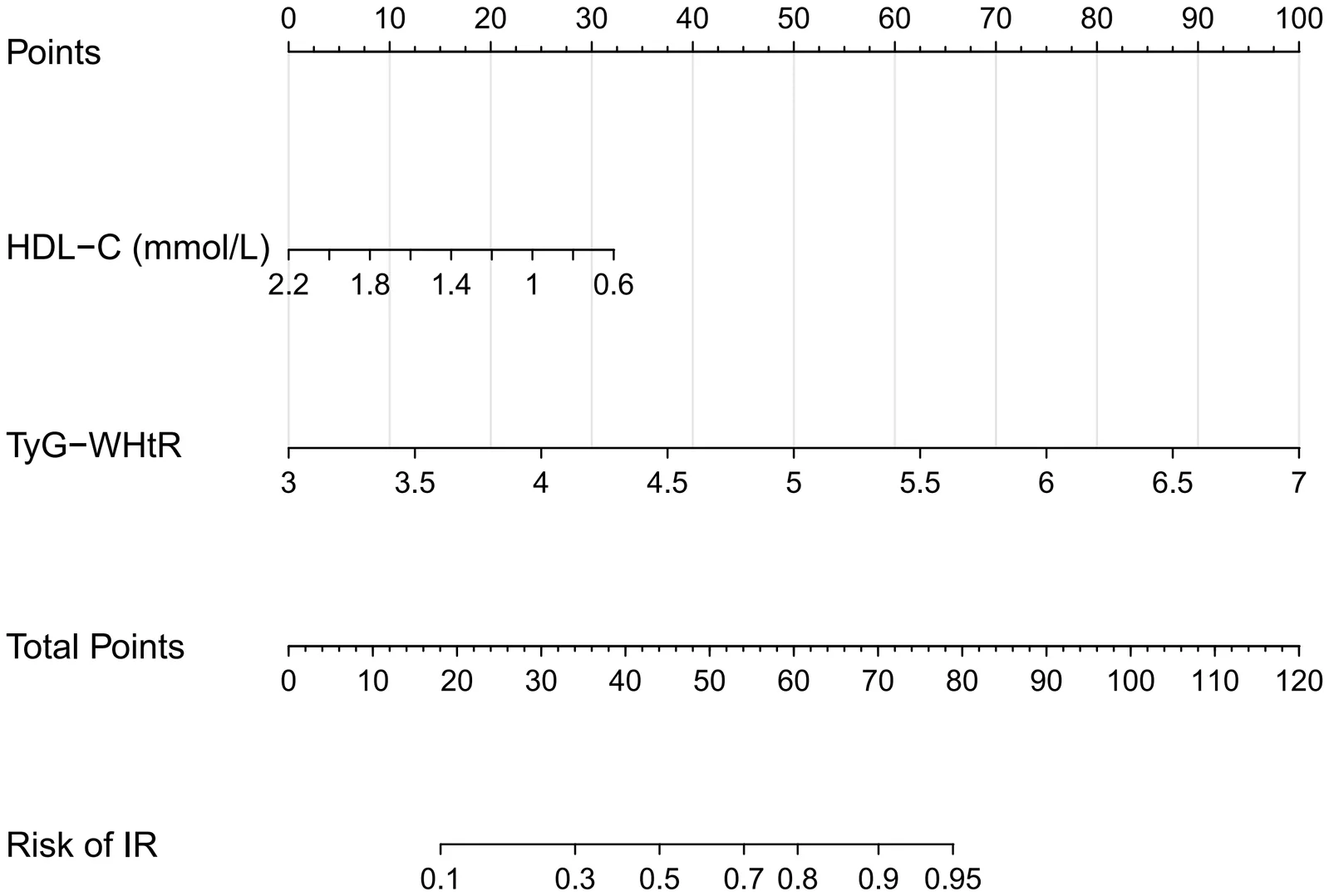
Nomogram association model for IR status in prediabetes.

#### 3.6.2 Model performance and clinical utility

The discriminative ability of the combined HDL-C and TyG-WHtR model was assessed by ROC analysis. In multivariable logistic regression, HDL-C was a protective factor (OR = 0.183, 95% CI 0.044–0.765, *P* = 0.020; B=−1.699). When analyzed alone, HDL-C yielded an AUC of 0.267 (95% CI 0.182–0.351, *P* < 0.001), which is below 0.5. Because an AUC below 0.5 indicates inverse prediction, the complementary AUC (0.733) was calculated, suggesting that this single marker still carried predictive information. To align the direction of risk across components of the composite marker, HDL-C was reverse-coded before inclusion in the combined model. The combined model achieved an AUC of 0.815 (95% CI 0.748–0.881, *P* < 0.001), outperforming TyG-WHtR alone (AUC = 0.793, 95% CI 0.722–0.864) and the reversed HDL-C (AUC = 0.733, 95% CI 0.649–0.818) (**Table 4D**, **Figure 2**).

**Table 4D:** ROC curve analysis: AUC, 95% CI, and optimal cut-off values.

Indicator	AUC	95%CI	*P*	Optimalcut-off value	Sensitivity	Specificity	Youden’sindex
TyG-WHtR	0.793	0.722–0.864	<0.001	4.61	0.491	0.906	0.397
HDL-C	0.733	0.649–0.818	<0.001	1.18	0.456	0.792	0.248
Logistic Regression Model	0.815	0.748–0.881	<0.001	0.44	0.842	0.472	0.314

HDL-C was reverse-coded for AUC.

**Figure 2:**
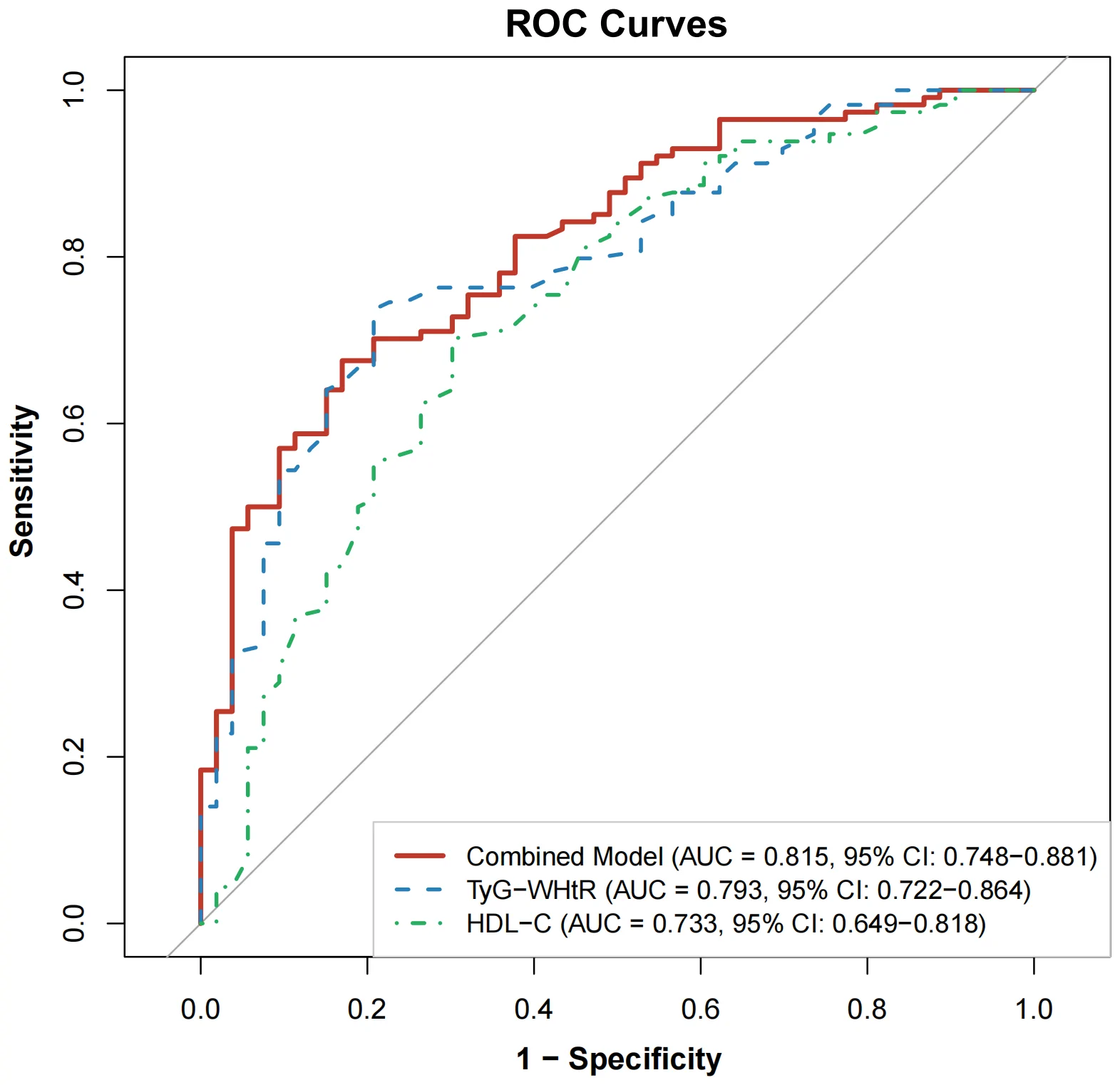
ROC curves for predicting IR. HDL-C was reverse-coded for AUC.

Model calibration was evaluated with the Hosmer-Lemeshow test and calibration curves; the C-index was 0.815 (95% CI 0.750–0.881). The apparent calibration curve lay close to the diagonal, and the bias-corrected curve closely overlapped the ideal line, with narrow 95% confidence bands after correction (**Figure 3**). Internal validation based on 1000 bootstrap resamples produced a bias-corrected calibration curve that nearly coincided with the ideal diagonal, indicating high agreement between predicted probabilities and observed event rates and no serious overfitting (**Figure 4**).

**Figure 3:**
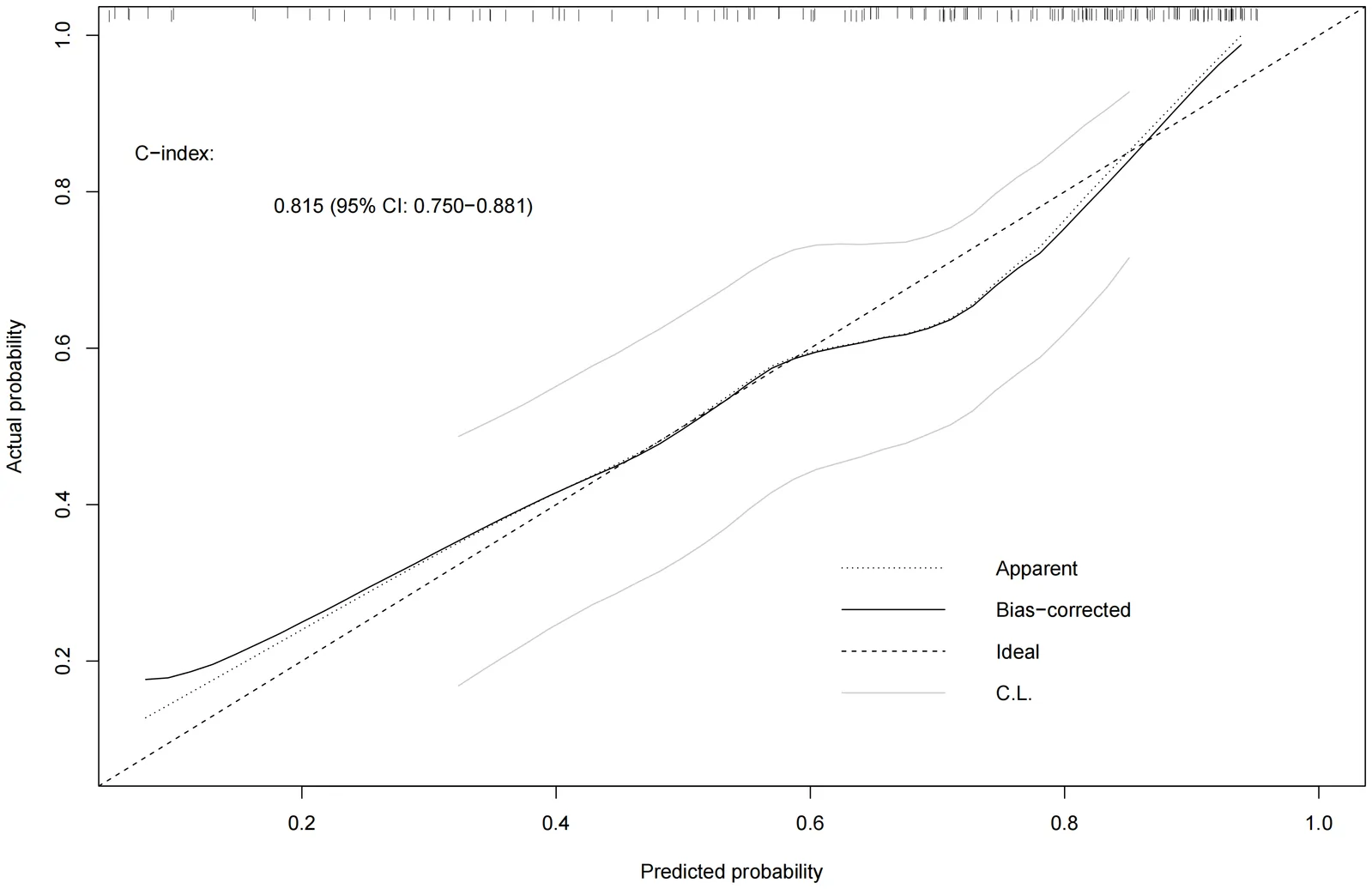
Hosmer-Lemeshow calibration curve for the nomogram predicting IR risk in prediabetes.

**Figure 4:**
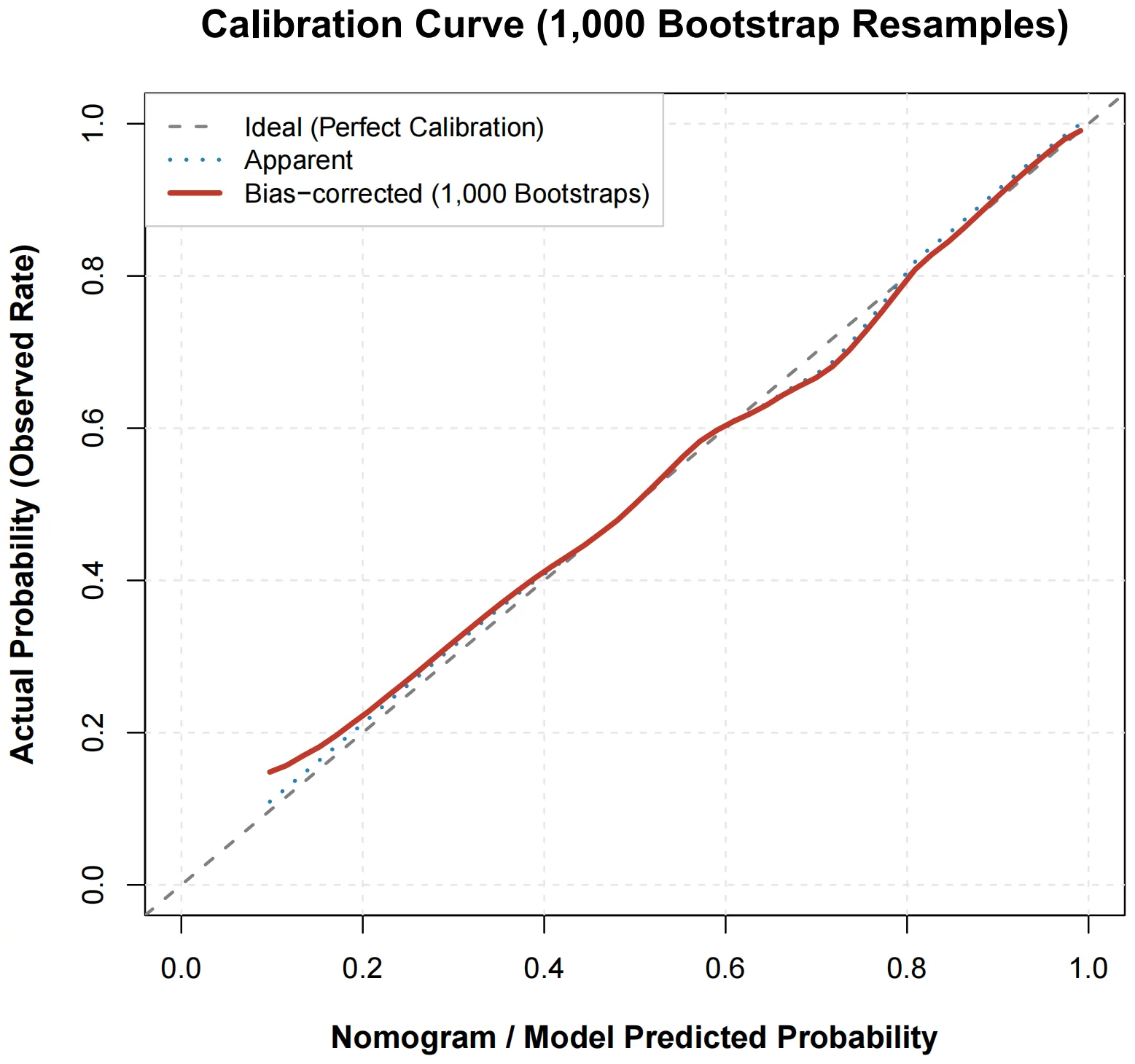
Bootstrap internal validation calibration plot of the nomogram model.

DCA further evaluated clinical net benefit (**Figure 5A**). For threshold probabilities between approximately 0.2 and 0.8, the standardized net benefit of the combined model remained consistently higher than that of the treat-all and treat-none strategies. Beyond a threshold of 0.9, the net benefit fluctuated owing to the reduced effective sample size, but the overall trend remained clear: the combined HDL-C and TyG-WHtR model offered net benefit for clinical risk stratification. The CIC quantified the intervention burden and event capture capacity (**Figure 5B**). As the high-risk threshold increased, the number of individuals classified as high risk per 1000 declined from nearly 1000 to 0, and the number of high-risk individuals who actually experienced events decreased steadily from the 600–800 range. Up to a cost-benefit ratio of approximately 3:2, the model maintained a high event capture rate. Fluctuations beyond a threshold of 0.8 were mainly attributable to limited sample size. Overall, the model retained its ability to identify key events while controlling overtreatment.

**Figure 5A:**
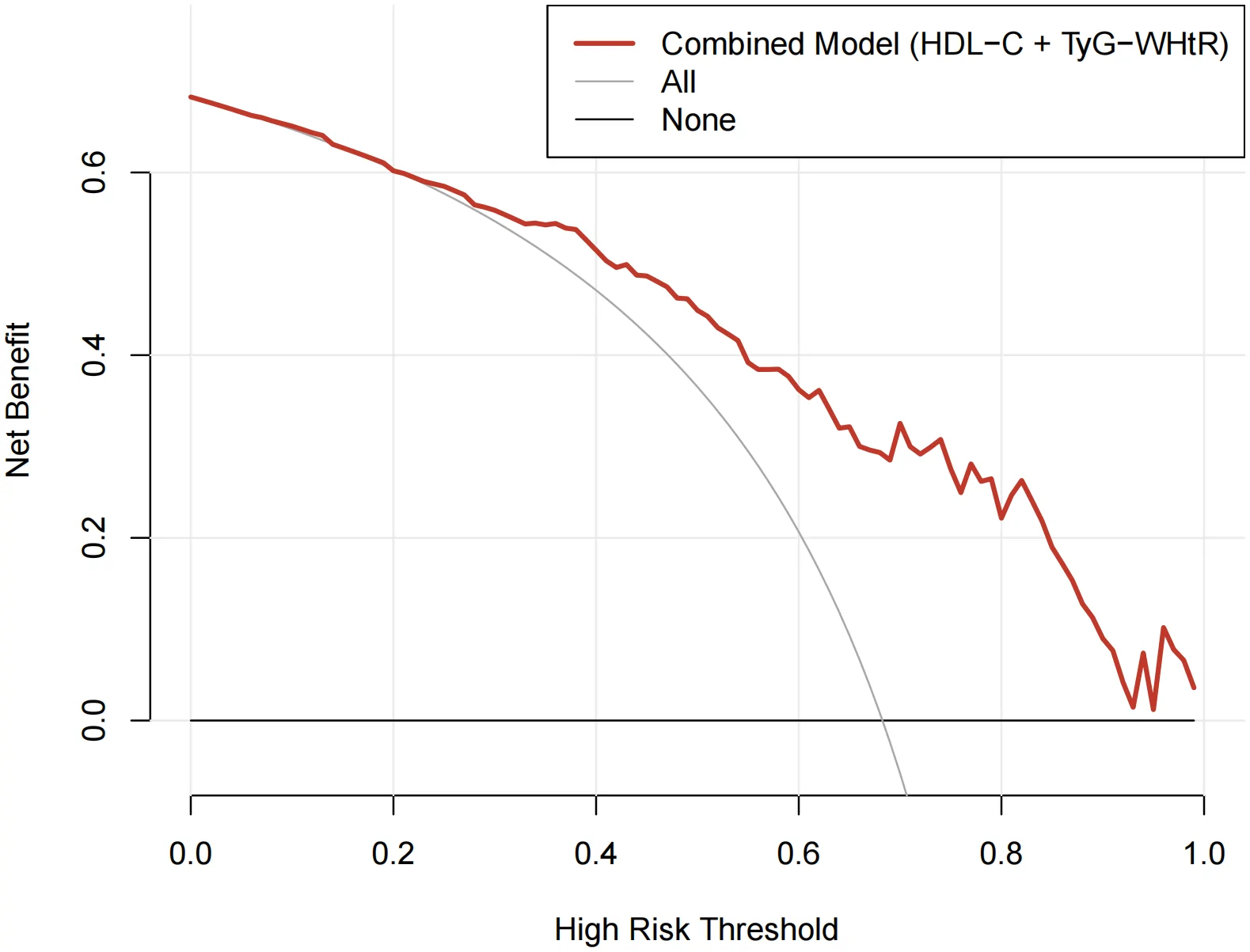
Decision curve of the nomogram for predicting insulin resistance risk in prediabetes.

**Figure 5B:**
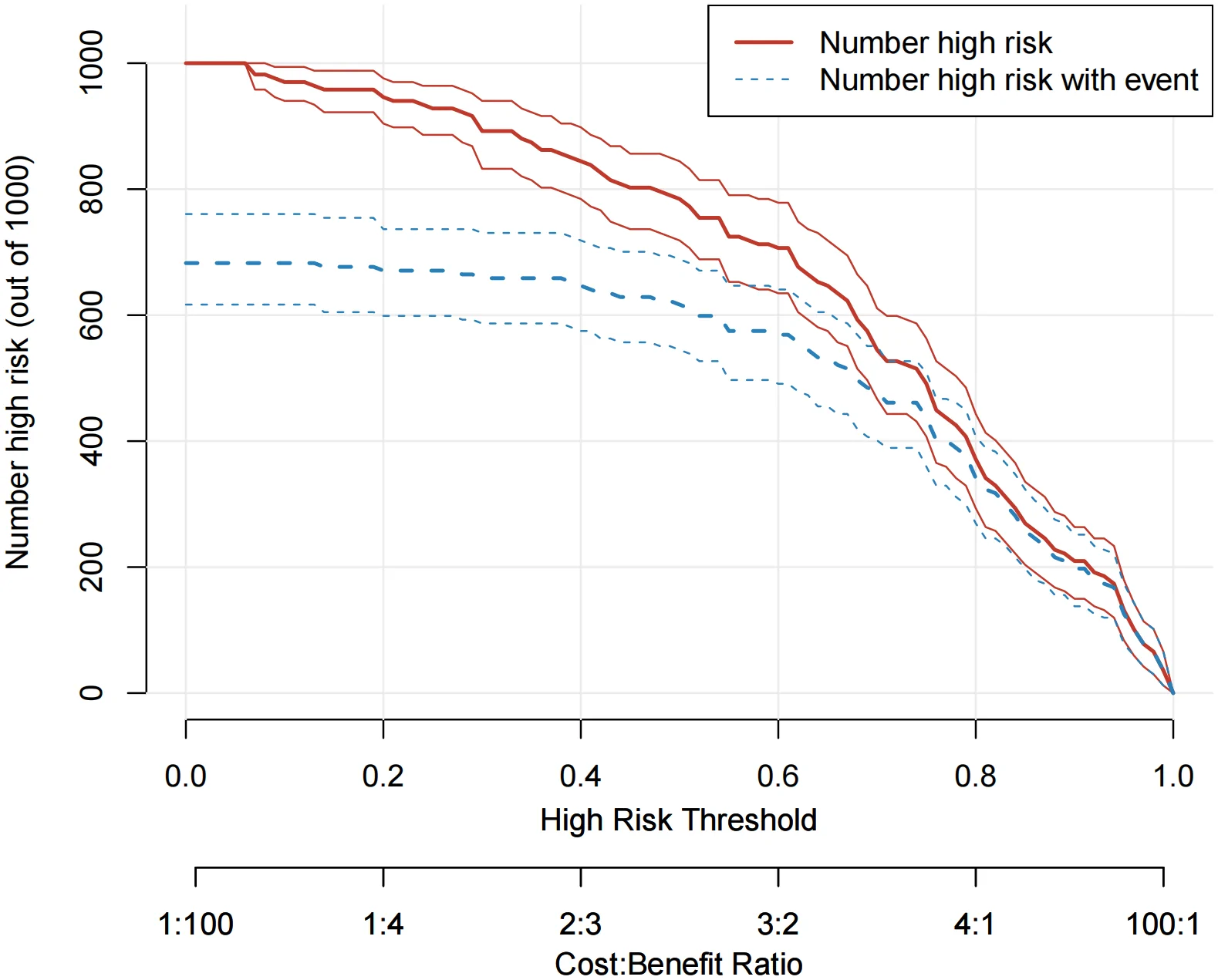
Clinical impact curve of the nomogram for predicting insulin resistance risk in prediabetes.

Taken together, these results provide mutually reinforcing evidence supporting the practical value of the combined model in clinical risk stratification.

### 3.7 Effect sizes of associated factors

We calculated the effect sizes of TyG-WHtR and HDL-C. According to Cohen’s criteria, d=0.2 is considered a small effect, d=0.5 a medium effect size, and d=0.8 a large effect. In the present study, the Cohen’s d for TyG-WHtR was 1.05 (95% CI, 0.71–1.40), and that for HDL-C was −0.90 (95% CI,−1.24 to −0.56), both representing large effects. These findings indicate that elevated TyG-WHtR and reduced HDL-C are strongly associated with insulin resistance and may hold potential clinical predictive value.

## 4 Discussion

A large-scale cohort study of over 100,000 Chinese adults has shown that individuals with overweight or obesity carry a substantially elevated risk of Pre-DM, with early warning signals emerging at a BMI as low as approximately 23.03 kg/m² (13, 14). Among those who had already developed Pre-DM, lower abdominal obesity and a lower TyG index were significantly associated with reversion to normoglycemia, indicating that reducing abdominal fat accumulation and ameliorating insulin resistance may serve as pivotal drivers for reversing the prediabetic state. Pre-DM complicated by obesity consistently conferred a higher risk of disease onset and progression, whereas Pre-DM accompanied by sarcopenia exhibited a bidirectional relationship: hyperglycemia in the prediabetic state frequently coexisted with sarcopenia, and sarcopenia, in turn, heightened the risk of progression from Pre-DM to type 2 diabetes mellitus (5, 15). This observation aligns with evidence demonstrating that Pre-DM is associated with reduced muscle mass, diminished muscle strength, slower gait speed, and severe sarcopenia (16), with these manifestations displaying notable sex-specific differences. When sarcopenia and obesity coexisted, the risk of Pre-DM was amplified to the highest level. Among non-elderly American adults, those with sarcopenic central obesity had an odds ratio of approximately 2.63 for developing Pre-DM (17). Based on the evidence outlined above, this study enrolled individuals with Pre-DM, examined their body composition and glucolipid metabolic profiles, and categorized them into four muscle–fat phenotypes according to muscle mass and visceral fat levels. We then investigated associations between these phenotypes and insulin resistance using multiplicative interaction tests and sex- and age-stratified analyses. Independent predictors were identified through multivariable logistic regression, and a nomogram model integrating TyG-WHtR and HDL-C was developed to clarify the role of combined muscle–fat status in insulin resistance, thereby informing the incorporation of body composition phenotypes into metabolic assessment and individualized risk stratification.

### 4.1 Body composition heterogeneity differentially drives metabolic risk

In the present study, with respect to glucose metabolism, FBG was inversely correlated only with ASMI, indicating that skeletal muscle mass is a key determinant of basal glucose homeostasis (18). In contrast, 2hPG showed inverse associations with BFM, PBF, and VFL. This apparent protective effect of adiposity on postprandial glucose likely reflects compensatory β-cell hypersecretion driven by adiposity-induced insulin resistance, whereas individuals with lower adiposity may have already lost this compensatory capacity owing to β-cell dysfunction (19). HbA1c was positively correlated only with WC and VFL, supporting the role of visceral fat as an independent marker of long-term diabetes risk (20). In terms of lipid metabolism, TG was broadly and positively correlated with all adiposity and muscle indices, confirming its status as the most sensitive lipid marker of energy excess (21). HDL-C showed consistent inverse associations with these indices, reflecting impaired HDL particle function in the setting of obesity (22). LDL-C was positively correlated with most body composition parameters, a pattern consistent with delayed LDL clearance mediated by downregulation of LDL receptors under insulin-resistant conditions (23). The TyG index and its derivatives were positively associated with all body composition variables, suggesting that these composite indices capture integrated information on fat accumulation and muscle metabolic burden and outperform individual lipid parameters in identifying early insulin resistance and metabolic risk (24). Consistent with this, Song K et al. demonstrated that TyG-WC and TyG-WHtR are superior to TyG alone in identifying metabolic syndrome (25). Integrating WHtR—a marker of central adiposity—into the TyG index likely amplifies its ability to detect insulin resistance. TyG-WHtR simultaneously captures abnormalities in triglyceride–glucose homeostasis and the pathological dimension of abdominal obesity, and may therefore more closely reflect the complex etiological network underlying insulin resistance.

Collectively, these findings provide mechanistic support for the utility of body composition–integrated indices: TyG derivatives such as TyG-WHtR, by incorporating information on fat accumulation (particularly central obesity) and muscle-related metabolic burden, may more comprehensively capture metabolic risk in individuals with Pre-DM.

### 4.2 The metabolic paradox of body composition: when muscle becomes a liability

This study reveals a counterintuitive phenomenon in Pre-DM: ASMI is positively correlated with HOMA-IR, defying the expected protective association. This paradox likely reflects the deeper mechanisms of the “obesity paradox.” Under conditions of energy surplus, skeletal muscle mass escapes net loss and may even increase in absolute terms due to heightened mechanical loading. Yet, myocellular insulin sensitivity and mitochondrial oxidative capacity decline markedly, coinciding with intramuscular fat accumulation (26)—a likely compensatory signature of ectopic lipid deposition and mitochondrial impairment. Therefore, an increase in muscle mass is not invariably synonymous with improved metabolic health.

This interpretation is further substantiated by the combined muscle-fat phenotyping analysis.

The HM/HF phenotype bears the greatest metabolic burden, validating Kim et al.’s observation that metabolic risk in high-fat phenotypes is largely triggered by dyslipidemia (10). Once adipose tissue storage capacity is saturated, increased lipolysis elevates circulating free fatty acids, driving ectopic deposition and culminating in systemic IR (27). In contrast, the LM/LF phenotype displays optimal insulin sensitivity, corroborating recent findings that muscle preservation amid low adiposity yields metabolic dividends (28, 29)—a pattern consistent with the lipid spillover hypothesis (30).Collectively, these results suggest that muscle’s metabolic value is inextricably linked to its surrounding lipid environment; thus, interpretation of muscle mass must be predicated on the concomitant adiposity status. When fat accumulation becomes the dominant driver, muscle may not only lose its buffering capacity but may even amplify insulin resistance.

Consequently, relying solely on body weight or BMI is insufficient. Integrating VFL and ASMI for muscle-adipose phenotypes enables a more granular assessment of IR risk.

### 4.3 Predominance of adiposity and clarification of the muscle–fat relationship

Hierarchical regression showed that, after adjustment for lipid parameters, the independent effect of the HM/HF phenotype on IR was attenuated but remained significant (B = 3.264, *P* < 0.001), whereas the protective effect of the LM/LF phenotype weakened to marginal significance (B=−0.705, *P* = 0.082). This graded pattern suggests that the association between low muscle mass and lower IR may be partly mediated by a more favorable lipid profile rather than reflecting an independent protective effect of low muscle mass itself. In other words, it is the low fat burden that likely confers the true protective effect, while muscle mass plays only a modifying role in this process.

Interaction analysis revealed no statistically significant multiplicative interaction between low muscle mass and high visceral fat (*P* = 0.089), arguing against the hypothesis that these two factors act synergistically to amplify IR in this study population. Taken together, these findings suggest that visceral fat accumulation is the dominant driver of IR, with muscle status exerting a secondary or conditional influence within this framework. This aligns with the work of Lingvay I, which posits that excessive fat accumulation is the fundamental upstream etiology of T2DM, and that the continuous expansion of fat mass is the core driver of IR (31).

### 4.4 Potential modifying effects of sex and age

The association between body composition phenotypes and IR may be influenced by sex and age. The HM/HF phenotype showed a particularly strong effect in men (B = 5.662, *P* < 0.001), whereas the effect was attenuated in women (B = 1.701, *P* = 0.002). The LM/LF phenotype retained an independent protective effect in women (B=−0.914, *P* = 0.025), although confounding by sample composition or other unmeasured factors cannot be excluded. This discrepancy may stem from the differential programming of body composition distribution and insulin sensitivity by sex hormones (19, 32).

The effect of the HM/HF phenotype was more pronounced among individuals younger than 60 years (B = 4.425,*P* < 0.001) but was no longer significant in those aged 60 years or older; in the latter group, only triglycerides remained a positive predictor (B = 0.545, *P* = 0.047). The sign of the sex coefficient even shifted from negative to positive in the ≥60-year group, although this did not reach statistical significance. This suggests that, in older adults, circulating lipid disturbances may replace body composition patterns as the more dominant factor associated with IR. Aging per se is not an independent driver of IR; rather, its effect is largely mediated by obesity, particularly central obesity (33). Larger prospective studies are warranted to clarify whether age genuinely modifies the association between body composition and IR.

### 4.5 Synergistic predictive utility of TyG-WHtR and HDL-C for IR and model validation

To further identify independent predictors of IR, forward stepwise logistic regression was performed after adjusting for covariates including age and sex. TyG-WHtR and HDL-C emerged as independent factors associated with IR. Specifically, HDL-C served as an independent protective factor (OR = 0.183,*P* = 0.020), exhibiting a negative association with IR. Under obese conditions, elevated cholesteryl ester transfer protein activity and enhanced hepatic lipase activity accelerate HDL catabolism, while insulin resistance suppresses apolipoprotein A-I synthesis, collectively contributing to reduced HDL-C levels (22). Conversely, TyG-WHtR demonstrated the strongest predictive power: each unit increase was associated with an 8.231-fold higher risk of IR (95% CI: 2.998–22.602, *P* < 0.001).

Meanwhile, a nomogram for predicting IR risk was successfully constructed and internally validated in this prediabetic population. ROC curve analysis showed that TyG-WHtR had high specificity (90.6%) but low sensitivity (49.1%), indicating that a positive TyG-WHtR result is more useful for ruling in IR, whereas a negative result cannot reliably exclude IR. In contrast, HDL-C alone had limited discriminatory capacity, consistent with its established role as a protective factor—its reduction primarily reflects underlying metabolic disturbances rather than serving as a direct diagnostic marker. However, when TyG-WHtR and HDL-C were jointly incorporated into the logistic regression model, the AUC improved to 0.815 (95% CI: 0.748–0.881, *P*<0.001), and sensitivity increased markedly to 84.2%, although specificity decreased to 47.2%. This high sensitivity makes the combined model more suitable for initial screening rather than confirmation: a negative result can help rule out IR, while a positive result should prompt further diagnostic evaluation. These findings align with those of Kim et al. (34), who reported that TyG-derived indices incorporating obesity measures may outperform TyG alone in discriminative ability. Our study extends this evidence by confirming the synergistic value of combining TyG-WHtR with HDL-C specifically in a prediabetic cohort.

Model calibration was evaluated using the Hosmer–Lemeshow test (*P*=0.650) and calibration curves. The C-index, which is equivalent to the AUC for a binary outcome, was 0.815 (95% CI: 0.748–0.881). After 1000 bootstrap resamples, the bias-corrected calibration curve closely approximated the ideal diagonal, with no clear evidence of overfitting on internal validation. Decision curve analysis indicated that the combined model provided a sustained net benefit over the treat-all and treat-none strategies across threshold probabilities from 0.2 to 0.8. The clinical impact curve showed that the model maintained high event capture with manageable intervention burden up to a cost-benefit ratio of approximately 3:2. These calibration, discrimination, and decision curve results were mutually consistent, supporting the combined HDL-C and TyG-WHtR model as a simple and well-calibrated tool for clinical risk stratification; this consistency also reduces the likelihood that any single metric reflects chance optimism. Nevertheless, independent external validation is still required.

At the optimal cut-off value (predicted probability ≥ 0.44), the combined model achieved a sensitivity of 84.2% and a specificity of 47.2%. Although the specificity is suboptimal, the high sensitivity helps minimize underdiagnosis, making it suitable as an initial screening tool in the primary care management of Pre-DM to identify high-risk individuals warranting lifestyle interventions or further diagnostic evaluation.

Several limitations of this study should be acknowledged. First, all body composition parameters were estimated by bioelectrical impedance analysis, the accuracy of which can be influenced by hydration status, electrode contact quality, recent food intake, and comorbid conditions; consequently, estimates of appendicular skeletal muscle mass index and visceral fat grade may deviate systematically from gold-standard measures obtained by MRI or CT. Second, the sample size was relatively limited, drawn from a single center, and the male subgroup was comparatively small, which may have reduced statistical power to detect certain associations. Larger multicenter studies are therefore needed to confirm these findings. Third, the cross-sectional design precludes causal inference, and because the model was not validated in an independent cohort, extrapolation of the results should be made with caution. Finally, future studies should incorporate inflammatory markers, adipokines, and measures of muscle strength and physical function to more accurately identify sarcopenia, validate the phenotype classification used here, and evaluate its long-term prognostic value in longitudinal cohorts.

## 5 Conclusion

This study suggests that Pre-DM is not a metabolically homogeneous condition, and BMI alone is insufficient to capture its internal heterogeneity. Using a combined muscle–fat classification based on ASMI and VFL, visceral adiposity emerged as the dominant driver of insulin resistance, while the metabolic effect of skeletal muscle depended heavily on the surrounding lipid milieu. The HM/HF phenotype carried the greatest metabolic burden, whereas the protective association of the LM/LF phenotype was almost entirely attributable to a lower visceral fat load. The interaction term between low muscle mass and high visceral fat did not reach statistical significance, and no significant multiplicative interaction was detected; additive interaction was not formally tested, and the study may be underpowered. Among metabolic indices, TyG-WHtR and HDL-C showed independent positive and negative associations, respectively, with notably large effect sizes. A combined nomogram incorporating these two indicators achieved an AUC of 0.815 with good calibration; however, its limited specificity suggests it is better suited as a preliminary screening tool in primary care. Exploratory stratified analyses indicated potential sex- and age-related differences: the risk associated with the HM/HF phenotype was particularly pronounced in men, while in individuals aged ≥60 years, triglycerides—rather than body composition—emerged as the predominant independent predictor. Overall, maintaining moderate muscle mass in the context of low visceral adiposity may confer greater metabolic benefit. Future studies should validate fixed phenotype cut-offs in independent cohorts and prospectively track progression to diabetes to assess the clinical net benefit of this model.

## Data Availability

The datasets presented in this article are not readily available because The data that support the findings of this study are available from the corresponding author upon reasonable request. The data are not publicly available due to privacy restrictions. Requests to access the datasets should be directed to Shujiao Chen, fjcsj12@163.com.
