# Supplementary material for: Discordant muscle-adipose phenotypes and insulin resistance in prediabetes: sex-specific associations and predictive utility of TyG-WHtR combined with HDL-C
Source: Front Endocrinol (Lausanne). 2026 Oct 15;17:1937778. doi: 10.3389/fendo.2026.1937778 (PMC13716647; doi:10.3389/fendo.2026.1937778)
Supplement: Supplementary file 1 [file SupplementaryFile1.pdf]

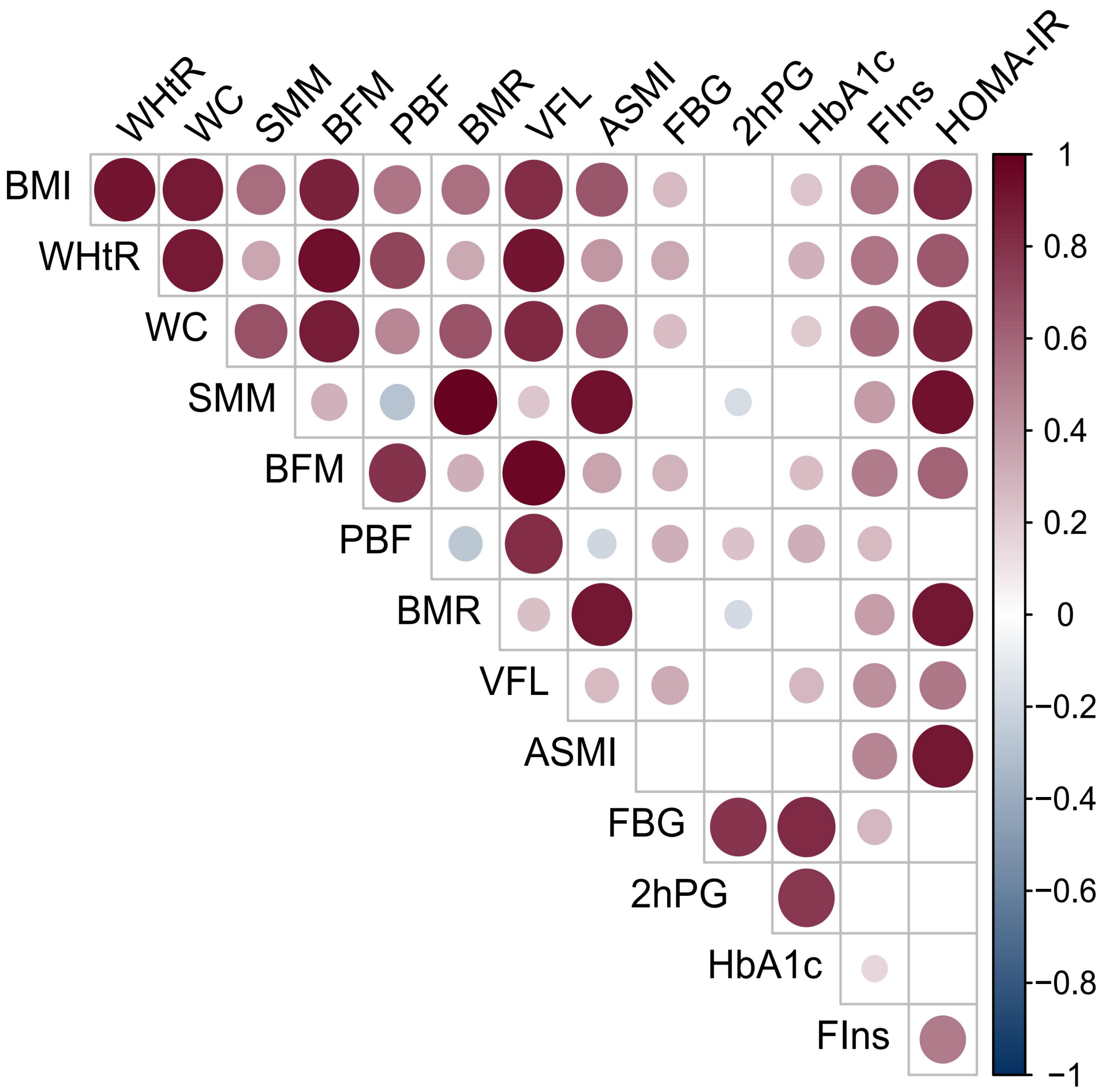

Note:Color represents Spearman's rho: red indicates a positive correlation and blue indicates a negative correlation; circle size represents |rho|; correlations with *P*>0.05 are automatically left blank.

Figure S1 Spearman correlation between glycemic metabolism markers and body composition

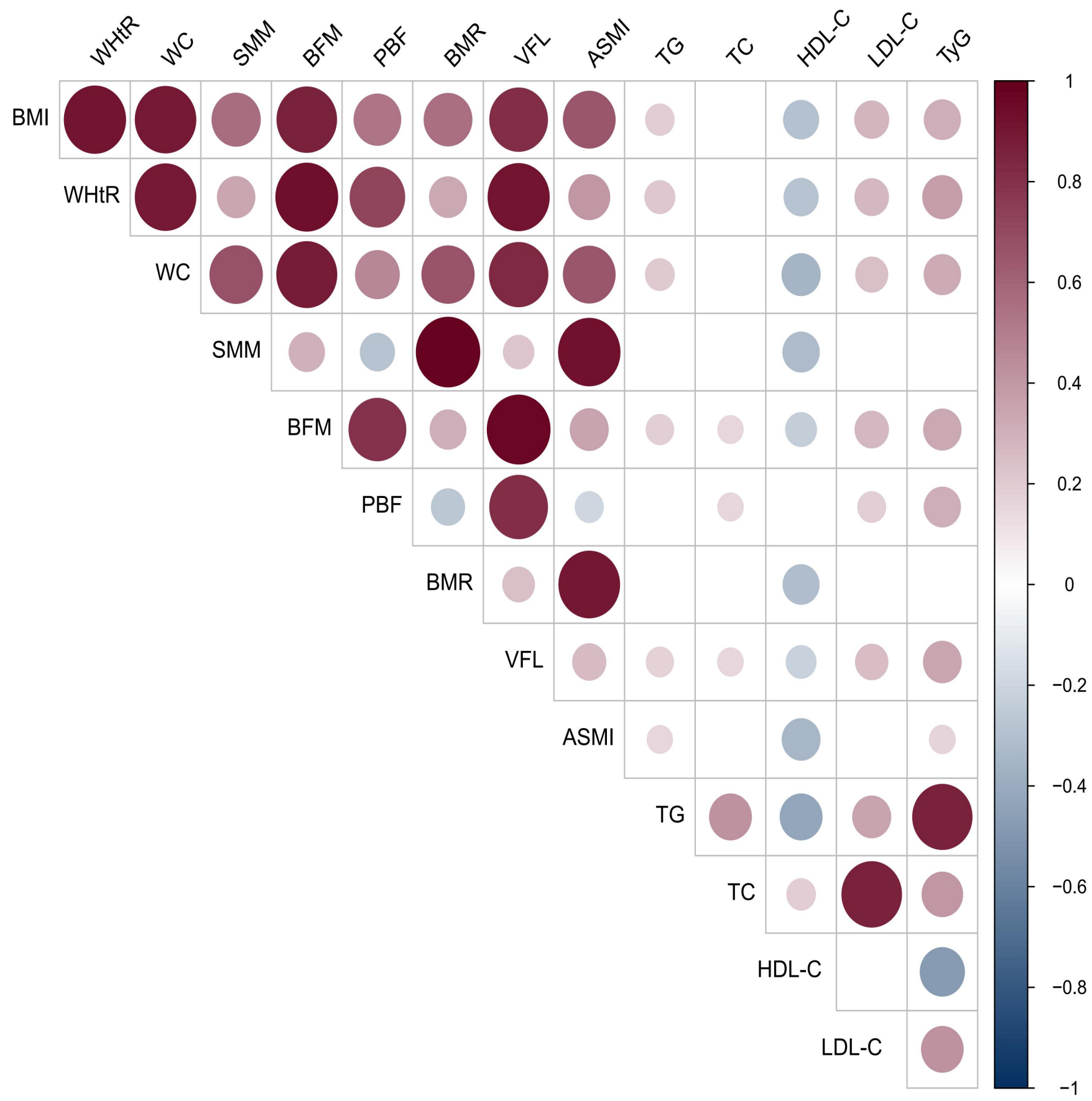

Note:Color represents Spearman's rho: red indicates a positive correlation and blue indicates a negative correlation; circle size represents |rho|; correlations with *P*>0.05 are automatically left blank.

Figure S2 Spearman correlation between lipid metabolic profiles and body composition

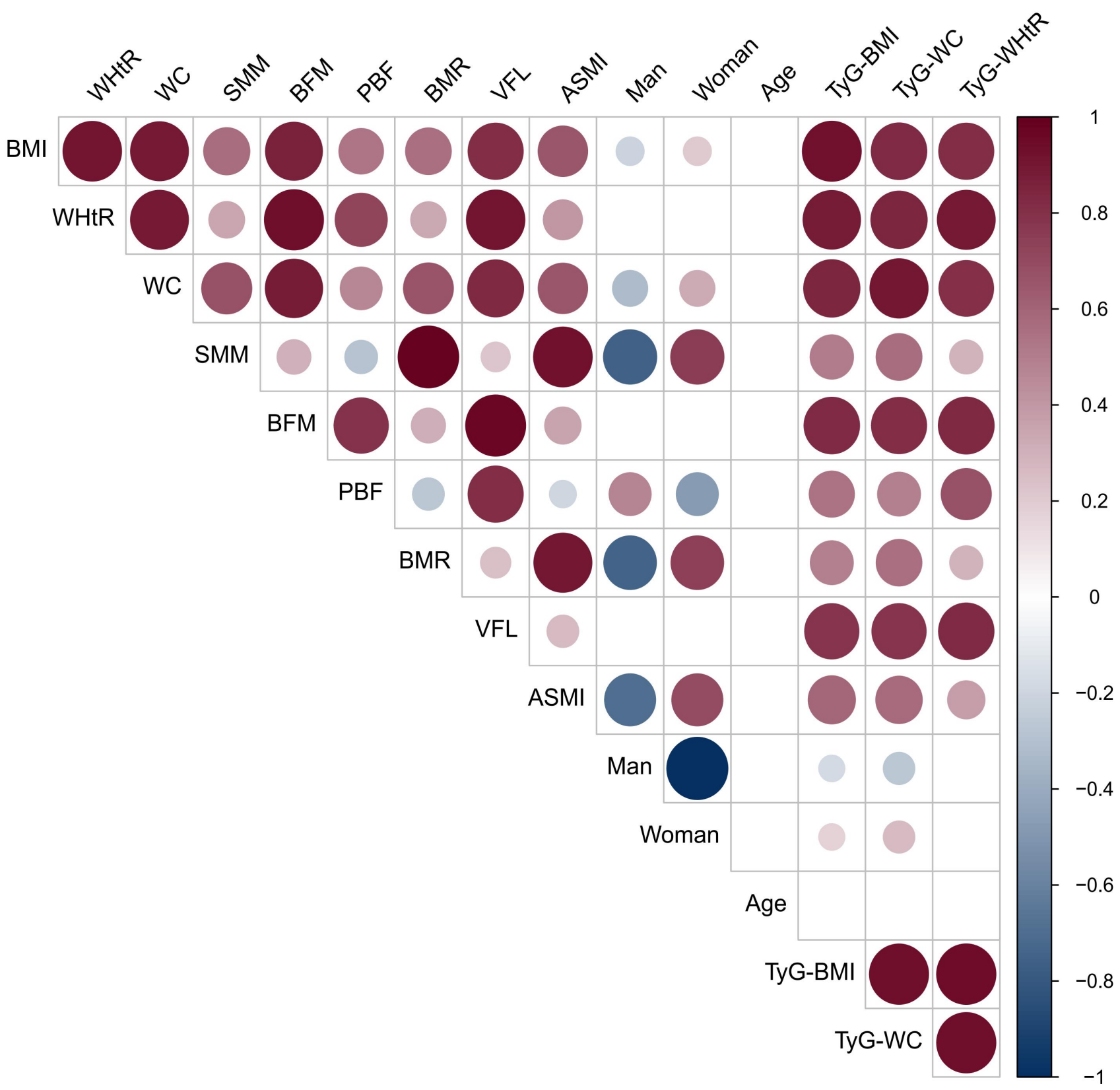

Note:Color represents Spearman's rho: red indicates a positive correlation and blue indicates a negative correlation; circle size represents |rho|; correlations with *P*>0.05 are automatically left blank.

Figure S3 Spearman correlation between TyG Index-derived metrics and body composition

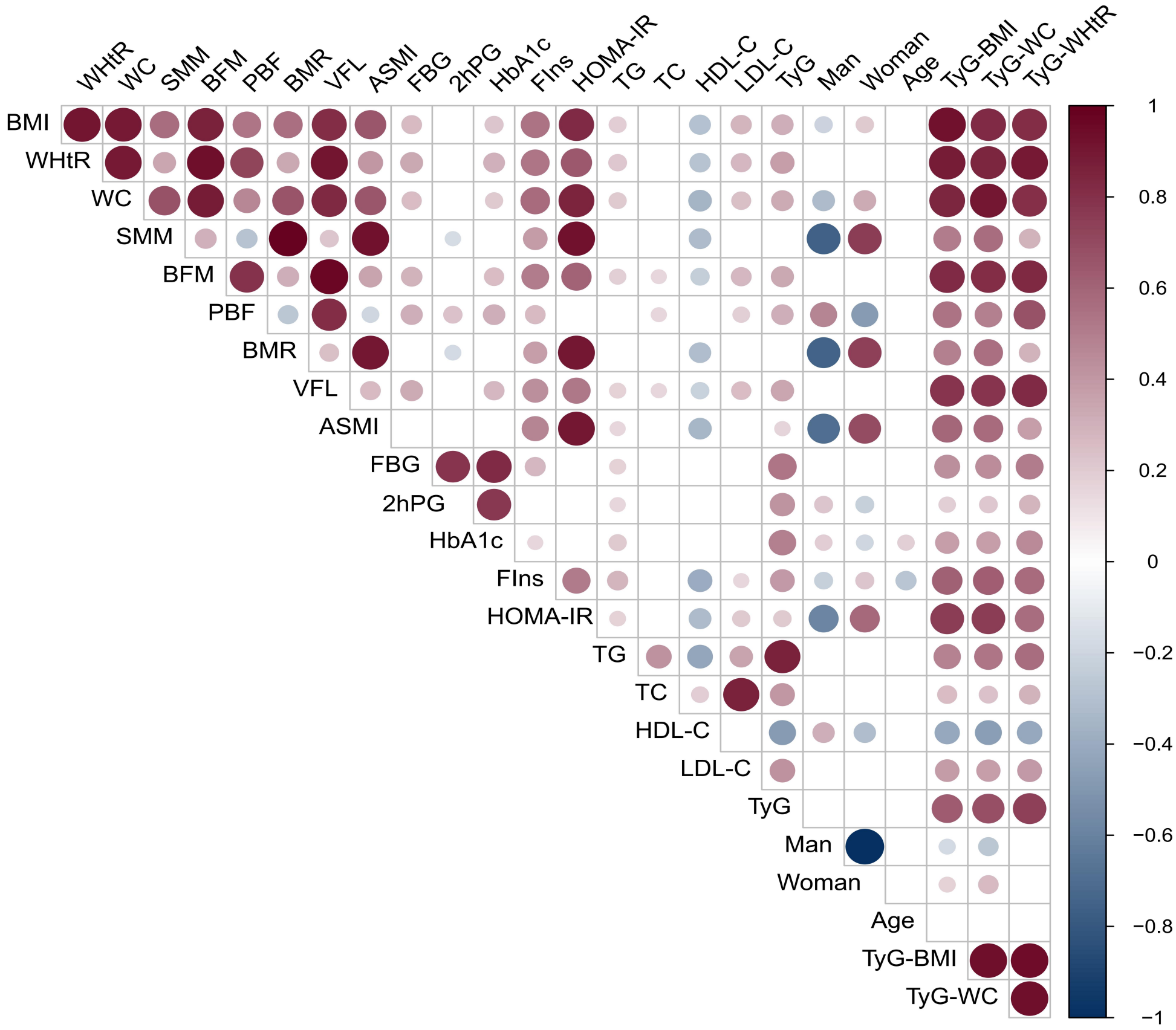

Note:Color represents Spearman's rho: red indicates a positive correlation and blue indicates a negative correlation; circle size represents |rho|; correlations with *P*>0.05 are automatically left blank.

Figure S4 Spearman correlation of HOMA-IR with clinical parameters

Tables S1 Comparison of body composition indices across different body composition phenotype groups (Mean ± SD / Median (Q1, Q3))

**Variable**	**HM/LF (n=71)**	**HM/HF (n=23)**	**LM/HF (n=12)**	**LM/LF (n=61)**	**H value**	***P* value**	**Adjusted *P* value**
BMI（kg/m²）	23.67（22.68,25.22）	28.76（26.27,31.56）	24.18（23.08,25.55）	21.66（19.70,22.62）	88.437	<0.001	G4<G1<G3<G2[1]
WC（cm）	82.20（79.20,86.70）	98.10（89.20,107.50）	85.60（82.50,89.85）	78.40（73.80,81.85）	68.881	<0.001	G4<G1<G3<G2[2]
WHtR	0.51（0.49,0.53）	0.59（0.57,0.63）	0.55（0.53,0.57）	0.48（0.46,0.50）	90.32	<0.001	G4<G1<G3<G2[3]
SMM（kg）	23.80（21.80,29.90）	27.10（22.30,32.80）	20.55（19.00,22.13）	21.10（19.55,25.50）	33.904	<0.001	G3、G4<G1、G2[4]
BFM（kg）	17.20（15.30,19.20）	25.70（22.90,29.10）	21.80（20.38,24.20）	15.70（13.15,17.40）	86.674	<0.001	G4、G1<G3、G2[5]
PBF（%）	27.70（24.20,29.90）	35.80（32.70,39.90）	36.60（35.65,38.98）	26.30（22.80,30.90）	68.133	<0.001	G4、G1<G2、G3[6]
BMR（kcal）	1320.00（1236.00,1526.00）	1424.00（1258.00,1627.00）	1198.00（1125.50,1265.25）	1212.00（1166.00,1387.50）	29.845	<0.001	G3、G4<G1、G2[7]
VFL	7.00（6.00,8.00）	12.00（10.00,14.00）	10.50（10.00,12.00）	6.00（5.00,8.00）	86.285	<0.001	G4、G1<G3、G2[8]
ASMI	6.90（6.60,7.90）	7.60（6.80,8.20）	6.00（5.93,6.18）	6.20（5.80,6.95）	62.648	<0.001	G3、G4<G1、G2[9]

Note:G1, HM/LF group; G2, HM/HF group; G3,LM/HF group; G4, LM/LF group. Data are presented as median (Q1, Q3). Group comparisons were performed using the Kruskal-Wallis H test, with post hoc pairwise analyses conducted via Dunn’s test. Pvalues were adjusted using the Bonferroni method.

Significance markers:[1]G4vsG1,G2:P<0.001;G4vsG3:P=0.001;G1vsG2:P<0.001.

[2]G4vsG1:P=0.004;G4vsG2:P<0.001;G4vsG3:P=0.001;G1vsG2:P<0.001.

[3]G4vsall:P<0.001;G1vsG2:P<0.001;G1vsG3:P=0.027.

[4]G4vsG1:P<0.001;G4vsG2:P=0.001;G4vsG3:P=0.002;G2vsG3:P=0.001.

[5],[6]G4vsG2,G3:P<0.001;G1vsG2,G3:P<0.001.

[7]G4vsG1:P<0.001;G4vsG2:P=0.001;G1vsG3:P=0.011;G2vsG3:P=0.004.

[8]Same pattern as in footnotes 5 and 6.

[9]G4vsG1:P<0.001;G4vsG2:P=0.001;G1vsG3,G2vsG3:P<0.001.

Tables S2　Comparison of glycometabolic profiles among phenotype groups　(Mean ± SD / Median (Q1, Q3))

Variable	HM/LF (n=71)	HM/HF (n=23)	LM/HF (n=12)	LM/LF (n=61)	H value	*P* value	Adjusted *P* value
FBG (mmol/L)	6.01 (5.47,6.80)	7.41 (5.59, 8.81)	6.12 (5.34, 9.21)	5.91 (5.36, 6.54)	8.348	0.039	-
2hPG (mmol/L)	8.20 (6.15,11.07)	9.13 (6.77, 12.07)	8.53 (7.05,14.43)	7.80 (6.52,10.09)	2.856	0.414	-
HbA1c (%)	6.40 (6.20,7.30)	7.00 (6.30,7.60)	6.40 (6.05,8.65)	6.30 (6.00,6.60)	9.037	0.029	-
FIns (mU/L)	12.10 (9.00,15.90)	15.60 (11.50,26.60)	16.00 (11.00,18.60)	9.10 (7.30,12.60)	29.765	<0.001	G4 vs G1;G4 vs G2;G4 vs G3[1]
HOMA-IR	3.38 (2.39,4.20)	5.38 (3.06,9.31)	4.61 (3.23,5.67)	2.51 (1.92,3.28)	37.172	<0.001	G4 vs G1;G4 vs G2;G4 vs G3;G1 vs G2[2]

Note: G1, HM/LF group; G2, HM/HF group; G3,LM/HF group; G4, LM/LF group. Data are presented as median (Q1, Q3). Group differences were assessed using the Kruskal-Wallis H test, with post hoc pairwise comparisons performed via Dunn's test. Pvalues were adjusted using the Bonferroni method.

Significance markers:

[1] Significant differences for G4 vs G1 (P=0.013), G4 vs G2 (P<0.001), and G4 vs G3 (P=0.010).

[2] Significant differences for G4 vs G1 (P=0.003), G4 vs G2 (P<0.001), G4 vs G3 (P=0.002), and G1 vs G2 (P= 0.039).

Tables S3 Comparison of lipid profiles among phenotype groups (Mean ± SD / Median (Q1, Q3))

Variable	HM/LF (n=71)	HM/HF (n=23)	LM/HF (n=12)	LM/LF (n=61)	H value	*P* value	Adjusted *P* value
TG（mmol/L）	1.14（0.89,1.68）	1.54（1.30,2.14）	2.06（1.22,2.30）	1.26（0.83,1.60）	12.529	0.006	-
TC（mmol/L）	5.05（4.32,5.73）	5.47（4.93,6.04）	5.27（4.99,5.79）	5.23（4.60,5.73）	3.772	0.287	-
HDL-C（mmol/L）	1.17（1.06,1.34）	1.12（0.99,1.24）	1.15（1.02,1.33）	1.35（1.12,1.53）	12.431	0.006	G4 vs G2[1]
LDL-C（mmol/L）	2.90（2.31,3.40）	3.30（2.95,3.72）	3.18（2.72,3.48）	2.82（2.54,3.31）	7.700	0.053	-
TyG	8.67（8.31,9.05）	9.08（8.78,9.48）	9.08（8.66,9.72）	8.67（8.30,8.99）	16.516	0.001	G4 vs G2;G4 vs G3;G1 vs G2;G1 vs G3[2]
TyG-BMI	208.00（195.25,217.95）	256.40（236.20,302.55）	226.25（206.30,236.60）	184.30（170.80,200.60）	77.925	<0.001	G4 vs G1; G4 vs G2; G4 vs G3; G1 vs G2[3]
TyG-WHtR	4.45（4.17,4.67）	5.42（5.17,5.92）	5.10（4.73,5.28）	4.17（3.92,4.45）	71.110	<0.001	G4 vs G1;G4 vs G2;G4 vs G3 ;G1 vs G2 ;G1 vs G3[4]
TyG-WC	716.81（687.45,746.39）	908.11（810.90,1003.49）	792.00（738.65,845.18）	674.44（633.40,725.29）	60.258	<0.001	G4 < G1 < G3 < G2[5]

Note: G1, HM/LF group; G2, HM/HF group;G3,LM/HF group; G4, LM/LF group. Data are presented as median (Q1, Q3). Group comparisons were performed using the Kruskal-Wallis H test, with post hoc pairwise analyses conducted via Dunn's test. *P* values were adjusted using the Bonferroni method.

Significance markers:

[1]G4 vs G2: P=0.016; all other comparisons were non-significant.

[2]G4 vs G2, G4 vs G3, G1 vs G2, and G1 vs G3: all $P<0.05$. G4 vs G1 and G2 vs G3: $P>0.05$ (non-significant).

[3]Specific Pvalues: G4 vs G1 ($P<0.001$); G4 vs G2 ($P<0.001$); G4 vs G3 ($P<0.001$); G1 vs G2 ($P<0.001$).

[4]Significant differences for G4 vs G1 ($P=0.026$), G4 vs G2 ($P<0.001$), G4 vs G3 ($P<0.001$), G1 vs G2 ($P<0.001$), and G1 vs G3 ($P=0.016$).

[5]Significant differences for G4 vs G2 ($P<0.001$), G4 vs G3 ($P<0.001$), and G1 vs G2 ($P<0.001$).
